# Effect of aspiration on the lungs in children: a comparison using chest computed tomography findings

**DOI:** 10.1186/s12887-019-1531-6

**Published:** 2019-05-22

**Authors:** Nobukazu Tanaka, Kanji Nohara, Akihito Ueda, Tamami Katayama, Miyuki Ushio, Nami Fujii, Takayoshi Sakai

**Affiliations:** 1 Division of Oral and Facial Disorders, Osaka University Dental Hospital, 1-8, Yamadaoka, Suita, Osaka, 565-0871 Japan; 2 Division of Functional Oral Neuroscience, Graduate School of Dentistry, Osaka University, Osaka, Japan; 3 Medical Corporation Toujinkai, Fujitate Hospital, Osaka, Japan; 4 Osaka Development Rehabilitation Center, Osaka, Japan

**Keywords:** Aspiration, Child, Computed tomography, Dysphagia, Pneumonia

## Abstract

**Background:**

Detecting and addressing aspiration early in children with dysphagia, such as those with cerebral palsy, is important for preventing aspiration pneumonia. The current gold standards for assessing aspiration are swallowing function tests, such as fiberoptic endoscopic evaluation of swallowing (FEES) and videofluorographic swallowing study; however, the relationship between aspiration of secretion vs aspiration of foodstuff and pulmonary injury is unclear. To clarify this relationship, we examined the correlations between pneumonia findings from chest computed tomography (CT) and the presence or absence of aspiration detected by FEES.

**Methods:**

Eighty-five children (11 years 2 months ±7 years 2 months) underwent FEES and chest CT. Based on the FEES findings, the participants were divided into groups: with and without food aspiration, and with and without saliva aspiration. Correlations between chest CT findings of pneumonia and the presence or absence of each type of aspiration were then examined.

**Results:**

No significant correlations were observed between food aspiration and chest CT findings of pneumonia, whereas saliva aspiration and chest CT findings of pneumonia were significantly correlated. In addition, saliva aspiration was significantly associated with bronchial wall thickening (*p* < 0.01) and atelectasis (*p* < 0.05).

**Conclusions:**

Our findings in children suggest that: (1) the presence or absence of food aspiration detected by FEES evaluation has little correlation with pneumonia, and (2) the presence or absence of saliva aspiration may be an indicator of aspiration pneumonia risk.

## Background

Children with diseases, such as cerebral palsy or diseases involving multiple disabilities, have swallowing impairment [1]. The prevalence of dysphagia is extremely high—from 85 to 89% [2, 3] to as high as 99% [4]—in children with severe cerebral palsy. Therefore, addressing dysphagia is an essential part of caring for children with disabilities.

Aspiration and aspiration pneumonia are the most important problems associated with dysphagia in children. Data from articles and clinical reports vary regarding the frequency of aspiration and aspiration-related pneumonia with aspiration reported in 21–79% of children with certain diseases or disabilities [5]. Moreover, most (60–100%) cases of aspiration involve silent aspiration [5]. In one study, the investigators found aspiration-related pneumonia in nearly half of the children with various illnesses who were hospitalized for pneumonia [6]. This finding and other data suggest a strong association between aspiration and pneumonia in children with disabilities [7–9].

Videofluoroscopy and videoendoscopy are the gold standard methods for detecting aspiration—in particular, silent aspiration—and are more precise than other clinical methods for assessing children [10]. In clinical practice, assessments by a videofluoroscopic swallowing study (VFSS) or by fiberoptic endoscopic evaluation of swallowing (FEES) are often used to determine how to address food aspiration. To prevent pneumonia, the recommended methods to address food aspiration include adjusting an individual’s posture when eating or changing to a diet with food textures (e.g., mush, minced, thickened with water) that are safer than those of foods that are aspirated, limiting oral intake, and stopping oral feeding [1, 11]. Thus, the aspiration of food is associated with pneumonia onset. However, studies of adults have not found a relationship between pneumonia and food aspiration detected by VFSS or FEES [12–14]. Furthermore, insufficient evidence exists demonstrating that limiting oral intake prevents pneumonia [15]. Therefore, deciding whether to limit oral intake based on the presence or absence of aspiration could result in overmanagement.

To examine the relationship between pneumonia in children and aspiration detected by a swallowing function test, we investigated the correlation between pneumonia findings in chest computed tomography (CT) and the presence or absence of aspiration, as detected by FEES that was conducted within 1 week of the CT examination.

## Methods

### Participants

The participants were 126 disabled children with severe motor disturbances and developmental retardation who received outpatient care for dysphagia at the Osaka Developmental Rehabilitation Center (Osaka, Japan) from 2012 to 2014 and who underwent FEES to evaluate swallowing function. Eighty-five children underwent FEES evaluations, after having undergone chest CT, as part of the standard medical practice, in the preceding week. They were included if their CT results could be analyzed. The exclusion criteria were as follows: (1) chest CT was administered more than 1 week before the FEES (36 participants) and (2) poor image quality due to body movements or other factors that made analysis difficult (5 participants).

The participants included 51 boys and 34 girls whose mean age was 11 years 2 months±7 years 2 months (age range, 11 months–26 years). Based on the criteria of the *International Classification of Diseases, 10th revision*, the children’s underlying diseases were categorized as cerebral palsy and other paralytic syndromes (74.1%), episodic and paroxysmal disorders (22.3%), chromosomal abnormalities not elsewhere classified (9.4%), congenital malformations of the nervous system (9.4%), diseases of the myoneural junction and muscles (5.9%), other disorders originating in the perinatal period (5.9%), and other diseases such as cerebrovascular accident and metabolic disorder (total, 11.8%). Many participants had concomitant diseases, the most common being cerebral palsy and epilepsy.

All participants received nutrition orally: by total oral intake in 43 (50.6%) children and by the combined use of tube feeding in 42 (49.4%) children. Pneumonia history was confirmed from medical records and other clinical notes.

### Procedure

#### Swallowing function test

The FEES was used as the swallowing function test [16, 17]. It involves a team approach, which entails the participation and cooperation of multiple disciplines. In particular, dentists, pediatricians, speech therapists, nurses, dental hygienists, and caregivers took part in the testing. The test was explained to the caregivers beforehand. For the test, the caregivers brought the food and eating utensils that the participants used regularly or daily. In addition, posture, feeding assistance, and other circumstances under which the participants regularly ate were reproduced as closely as possible for the test. The participants were fed the test food by a caregiver or speech therapist. The test food was colored beforehand with green food dye to make it easy to visualize during the assessment. On conducting the test, the absence of fever, low oxygen saturation, fatigue, or other acute symptoms were confirmed in all patients.

After inserting the endoscope, it was manipulated to a low position so that the vestibule of the larynx was in the center of the field of view. After observing the larynx to check for saliva aspiration, the test food was ingested to continue the test. At least two mouthfuls of each texture of the test food were ingested. Aspiration was assessed using the penetration-aspiration scale (PAS), which is the standard assessment criterion for FEES for food [18]. Participants who cried when the fibroscope was inserted or who could not be evaluated because of hypertonia were excluded (3 participants). Videos of the tests were viewed by a dentist, pediatrician, and speech therapist. Based on the results, the participants were placed into the aspiration group (PAS score of 5–8) or the nonaspiration group (PAS score of 1–4). The children were then categorized into the food aspiration group or the saliva aspiration group.

### History of pneumonia

History of pneumonia was confirmed, based on the presence or absence of pneumonia in the patient’s medical records in the year before the FEES. Patients with this information in their records were considered as having a history of pneumonia.

### Chest CT

Chest CT images were obtained using the Asteion TSX-021B CT scanner (Toshiba Medical Systems, Tokyo, Japan). The imaging parameters were 120 kVp, 1.375:1 pitch, and an automatically adjusted current of 100–150 mA. Axial images were acquired at 2-mm slice thickness.

The CT images were assessed by a respiratory organ specialist (A.U.) and pediatrician (T.K.). They had more than 20 years of clinical experience and were blind to the FEES results. When their assessments did not match, they both re-evaluated the images and discussed them until a consensus was reached. When assessment of an image was difficult because of artifacts in the images resulting from a patient’s inability.

to control body movement during imaging, the participants were excluded (*n* = 5). Based on the method described by Butler et al. [14], the images were assessed for parenchymal bands, bronchiolectasis, bronchial wall thickening, bronchiectasis, atelectasis, tree-in-bud pattern, intraluminal airway debris, fibrosis, and air trapping. Participants with any of these CT findings were considered as having pneumonia.

### Data analysis

The aspiration and nonaspiration groups were compared for history of pneumonia and chest CT findings of pneumonia.

### Statistical analysis

Correlations between aspiration and history of pneumonia and between aspiration and CT findings of pneumonia were examined using the chi-square test or Fisher’s exact test. The influence of aspiration on CT findings of pneumonia was examined using logistic regression analysis in which aspiration was the independent variable and the various CT findings of pneumonia were the dependent variables. Forced entry of age and sex as the moderator variables was used for multivariate analysis. All tests were two-tailed. The significance level was *p* < 0.05. Missing values were not supplemented in the analysis. Outlier and extreme values were not excluded. Data were analyzed using SPSS version 22.0 for Windows (IBM Japan, Tokyo, Japan).

## Results

### Aspiration and history of pneumonia

Among 85 participants, the FEES findings revealed food aspiration in 48 (56.5%) participants, saliva aspiration in 26 (30.6%) participants, and both food and saliva aspiration in 20 (23.5%) participants. Among 54 participants with aspiration, 40 (74.1%) participants had silent aspiration. History of pneumonia within 1 year was observed in 33 (38.8%) participants.

### Chest CT findings

The CT image analysis revealed signs of pneumonia in 54 (63.5%) of 85 participants. The CT image findings were parenchymal bands (six [7.1%] participants), bronchiolectasis (27 [31.8%] participants), bronchial wall thickening (46 [54.1%] participants), bronchiectasis (two [2.4%] participants), atelectasis (17 [20.0%] participants), tree-in-bud pattern (eight [9.4%] participants), intraluminal airway debris (four [4.7%] participants), and other findings (four [4.7%] participants).

In the food aspiration/nonfood aspiration groups, 5/1 (10.4%/2.7%) participants had parenchymal bands; 13/14 (27.1%/37.8%) participants, bronchiolectasis; 27/19 (56.3%/51.4%) participants, bronchial wall thickening; 1/1 (2.1%/2.7%) participant, bronchiectasis; 11/6 (22.9%/16.2%) participants, atelectasis; 4/4 (8.3%/10.8%) participants, tree-in-bud pattern; 4/0 (8.0%/0%) participants, intraluminal airway debris; and 2/2 (4.0%/5.9%) participants, other findings.

In the saliva aspiration/non-saliva aspiration groups, 2/4 (7.7%/6.8%) participants had parenchymal bands; 11/16 (42.3%/27.1%) participants, bronchiolectasis; 20/26 (76.9%/44.1%) participants, bronchial wall thickening; 0/2 (0/3.4%) participants, bronchiectasis; 9/8 (34.6%/13.6%) participants, atelectasis; 1/7 (3.9%/11.9%) participants, tree-in-bud pattern; 3/1 (11.5%/1.7%) participants, intraluminal airway debris; and 3/1 (11.5%/1.7%) participants, other findings (Table 1).

**Table 1:** Chest CT findings as indicators of aspiration status

	Food Aspiration		Saliva Aspiration	
	Aspirator (*n* = 48)	Nonaspirator (*n* = 37)	Aspirator (*n* = 26)	Nonaspirator (*n* = 59)
Chest CT findings				
Parenchymal band	5(10.42)	1(2.70)	2(7.69)	4(6.78)
Bronchiolectasis	13(27.08)	14(37.84)	11(42.31)	16(27.12)
Bronchial wall thickening	27(56.25)	19(51.35)	20(76.92)	26(44.07)
Bronchiectasis	1(2.08)	1(2.70)	0(0)	2(3.39)
Atelectasis	11(22.92)	6(16.22)	9(34.62)	8(13.56)
Tree-in-bud pattern	4(8.33)	4(10.81)	1(3.85)	7(11.86)
Intraluminal airway debris	4(8.33)	0(0)	3(11.54)	1(1.69)
Other findings	2(4.17)	2(5.41)	3(11.54)	1(1.69)

The data are presented as the number (percentage) of participants.

CT, computed tomography.

### Correlation between aspiration and history of pneumonia and between aspiration and chest CT findings of pneumonia

A history of pneumonia, based on medical records from the previous year, was not correlated with the presence or absence of food or saliva aspiration (Table 2; *p* = 0.545 and *p* = 0.964, respectively). Food aspiration and chest CT findings of pneumonia were not correlated (*p* = 0.120). However, a significant correlation was observed with saliva aspiration (Table 3, *p* < 0.01). Logistic regression analysis revealed that saliva aspiration had a significant effect on bronchial wall thickening (odds ratio [confidence interval]: 4.231 [1.485–12.055]) and atelectasis (odds ratio [confidence interval]: 3.375 [1.124–10.131]). Multivariate logistic regression analysis with age and sex included as the moderator variables revealed similar effects. By contrast, food aspiration did not have a significant effect on any finding (Table 4).

**Table 2:** Comparison of the history of pneumonia between aspirators and non-aspirators

	Food aspiration		p-value		Saliva aspiration		*p*-value	
	Aspirator (n = 48)	Non-aspirator (n = 37)			Aspirator (n = 26)	Non-aspirator (n = 59)		
Pneumonia								
Yes	20 (41.67)	13 (35.14)	0.545^a^	0. 655^b^	10 (38.46)	23 (38.99)	0.964^a^	> 0.999^b^
No	28 (58.33)	24 (64.86)			16 (61.54)	36 (61.01)		

The data are presented as the number (percentage) of participants.

^a^The *p*-value, based on the Pearson’s chi-squared test

^b^The *p*-value, based on the Fisher’s exact test

**Table 3:** Comparison of chest CT findings between aspirators and non-aspirators

	Food aspiration		p-value		Saliva aspiration		p-value	
	Aspirator (n = 48)	Non-aspirator (n = 37)			Aspirator (n = 26)	Non-aspirator (n = 59)		
Pneumonia								
Yes	34 (70.83)	20 (54.05)	0.111 ^a^	0.120 ^b^	23 (88.46)	31(52.54)	0.002^a^	> 0.001^b^
No	14 (29.17)	17 (45.95)			3 (11.54)	28 (47.46)		

The data are presented as the number (percentage) of participants.

^a^The p-value, based on the Pearson’s chi-squared test

^b^The *p*-value, based on the Fisher’s exact test

CT, computed tomography; RC, regression coefficient; OR, odds ratio; 95% CI, 95 confidence interval; n.c., not calculated.

**Table 4:** Relationship between saliva aspiration and chest CT findings

	Univariate logistic regression analysis				Multivariate logistic regression analysis (adjusted for age and sex)			
	RC	OR	95% CI	p-value	RC	OR	95% CI	p-value
Parenchymal band	0.136	1.146	0.196–6.685	0.880	0.206	1.229	0.202–7.465	0.823
Bronchiolectasis	0.678	1.971	0.749–5.182	0.169	0.687	1.987	0.737–5.361	0.175
Bronchial wall thickening	1.442	4.231	1.485–12.055	0.007	1.645	5.182	1.663–16.151	0.005
Bronchiectasis	n.c.				n.c.			
Atelectasis	1.216	3.375	1.124–10.131	0.030	1.210	3.353	1.088–10.334	0.035
Tree-in-bud pattern	−1.214	0.297	0.035–2.548	0.268	−1.236	0.291	0.034–2.501	0.261
Intraluminal airway debris	2.024	7.565	0.748–76.507	0.087	1.961.	7.107	0.673–75.020	0.103

CT, computed tomography; RC, regression coefficient; OR, odds ratio; 95% CI, 95 confidence interval; n.c., not calculated.

## Discussion

### Assessment with FEES

We used FEES to assess aspiration in this study. The gold standard methods for evaluating swallowing functions are FEES and VFSS. Their detection rates of aspiration do not have a significant error [19, 20], and they are useful in children and adults [21–23]. In our findings, 56% of the tested participants had aspiration, which did not substantially differ from the results in previous research [1]. Furthermore, 74% of the participants with aspiration exhibited silent aspiration. This finding is consistent with that of another report [5] demonstrating that silent aspiration was common among patients with aspiration. This finding in the present study indicated that using an FEES study is valid for assessing aspiration.

A contrast agent is unnecessary in FEES; therefore, food that is eaten normally can be used as the test food. Another major advantage of FEES is that aspiration can be confirmed, despite the absence of contrasted saliva, secretions, or other matter. Our results showed that, compared with food aspiration, saliva aspiration detected by FEES was correlated with inflammatory findings in the lungs. This finding indicated that FEES, which is able to assess saliva aspiration, is a useful swallowing function test.

### CT assessments

In the present study, CT was used to evaluate inflammatory findings in the lungs; CT involves more radiation exposure and takes longer to conduct than does chest radiography and requires that body movements be controlled for a certain amount of time. Despite these issues, CT can detect dorsal and asymptomatic inflammatory findings. On account of its superior ability in detecting early stage lung changes and inflammatory findings, we considered it as the optimal method to carefully examine the relationship between aspiration and chest findings. Furthermore, for the current study, chest CT was conducted in a medical center that specializes in the care of disabled children. This center has specialized staff who are accustomed to interacting with disabled children and technicians who routinely image such patients. Therefore, except for the few patients who were excluded, we obtained images with sufficient precision for the assessments.

### Pneumonia history and food aspiration detected by FEES

A history of pneumonia in the previous year was not correlated with the presence or absence of food or saliva aspiration. Weir et al. [24] examined the relationship between pneumonia history and the presence or absence of food aspiration (detected by VFSS) in children. The authors found a low correlation between food aspiration detected by VFSS and history of pneumonia, based on the World Health Organization definition, in the year before VFSS [24]. We used FEES but not VFSS to evaluate aspiration, but our results were similar to those of Weir et al. [24]. However, pneumonia was determined based on clinical symptoms such as fever or confirmed in medical records; therefore, a variation in the diagnoses could have affected the accuracy of the assessments.

Furthermore, because the time difference between the aspiration assessment and the onset of pneumonia was as much as 1 year, the assessments could have been affected by other factors that may have appeared between the onset of pneumonia and the assessment (e.g., a food-related intervention or onset of a different disease). Therefore, to examine the relationship between aspiration detected during testing and pneumonia, we used chest CT findings, which are associated with little diagnostic variation, and FEES performed within 1 week after CT.

### Food aspiration and chest CT findings of pneumonia

In the present study, we examined the relationship between the presence or absence of aspiration detected by FEES, and the presence or absence of pulmonary inflammation in the chest CT image obtained within 1 week before FEES (inclusive of the day of the test). Compared with findings in previous studies [6, 7, 9, 12–14, 24] that examined aspiration (detected by testing) and history of pneumonia, the present study had little time difference between the swallowing function test and the chest evaluation, which indicated the swallowing function at the time of the test was compared with the state of the lungs. Thus, food aspiration detected by testing was not correlated with inflammatory findings on chest CT. The study by Butler et al. [14] involved an older population and CT timing, and revealed no correlation between food aspiration detected by FEES and inflammatory findings in chest CT conducted within 1 year of the test. Our findings were derived from children; however, they support the findings of Butler et al. [14]. Thus, our findings suggested that food aspiration detected during testing does not necessarily represent a risk of pneumonia in disabled children. In addition, regarding food aspiration, our findings suggested that it is necessary to consider not only the presence or absence of aspiration, which is the conventional evaluation standard, but also the amount and frequency of aspiration.

### Saliva aspiration and chest CT findings of pneumonia

We observed a significant correlation between saliva aspiration and chest CT findings of pneumonia. Furthermore, in a multivariate logistic regression analysis using age and sex as the moderator variables, saliva aspiration detected during testing was significantly correlated with the chest CT findings of bronchial wall thickening and atelectasis. Thus, CT may detect inflammatory changes of the lungs at an early stage. We observed atelectasis and bronchial wall thickening, which presumably occur because of repeated aspiration and inflammation of the bronchi caused by aspiration [25, 26]. In the present study, we used the advantages of FEES to examine, without contrast, the aspiration of food or other secretions such as saliva in the pharynx. Furthermore, to minimize stimulation of the oral cavity induced by test food ingestion or by inserting the endoscope, saliva aspiration was immediately assessed after inserting the endoscope and before the ingestion of the test food. Therefore, saliva aspiration detected by the test may reflect a chronic decrease in swallowing function or may reflect regularly occurring aspiration. In fact, 77% of participants who exhibited saliva aspiration also had food aspiration. Link et al. [27] reported that larger secretion pools were associated with an increased incidence of aspiration pneumonia in children. In another study [28], the researchers demonstrated that the extent of the pooling of secretions in the laryngopharynx was predictive of aspiration. These findings indicated that the presence or absence of saliva aspiration detected immediately after endoscope insertion could be used to predict pneumonia risk.

In the present study, the PAS was used as the assessment criterion for swallowing function, along with FEES and VFSS. However, this scale evaluates the presence or absence of aspiration from a few mouthfuls of test food ingested at the test venue; thus, it does not reflect the amount of aspiration normally present, the content of aspirated matter (i.e., apparent or silent), or the possibility of expectorating the aspirated matter. The PAS is an excellent indicator of swallowing function; however, it may be insufficient when considering pneumonia as an outcome. It may also be necessary to evaluate saliva aspiration, as well as the test food. The findings of the present study indicated that the management of swallowing dysfunction that is based only on the presence of food aspiration may result in excessive restriction or stopping of oral feeding, which may not allow an individual to maintain or develop swallowing function or may result in overmanagement that harms an individual’s food-related quality of life. Further investigations focusing on how to interpret swallowing function tests are needed.

### Limitations

The participants of this study were children with developmental disabilities who were unable to understand or follow instructions during CT. Therefore, it was impossible for them to control their body movements or maintain maximum inspiration during imaging. The imaging conditions were less than ideal; therefore, unrevealed findings may exist. To address this possibility, we excluded five participants whose images contained artifacts because of body movement or were otherwise difficult to interpret. By only evaluating participants whose images the assessors could interpret, we avoided lowering the precision of the image evaluations as much as possible.

In this study, it could not be determined whether the pneumonia was caused by aspiration or community acquired-infection. It was difficult to accurately diagnose whether the pneumonia was due to aspiration. However, 1) all subjects had swallowing impairment; and 2) all CT findings examined in this study were inflammatory findings due to aspiration. Based on these findings, the probability of pneumonia having been caused by aspiration is quite high in the subjects of this study.

In order to avoid missing saliva aspiration as much as possible, measures were taken, such as securing an appropriate field of view at the time of evaluation and confirmation of findings by multiple evaluators. However, the possibility of missing saliva aspiration is a limitation of this study.

We examined whether the presence or absence of aspiration during testing was associated with chest CT findings of inflammation and history of pneumonia in disabled children. Pneumonia history was not correlated with the presence or absence of food aspiration or with pulmonary CT findings. By contrast, the presence of saliva aspiration was significantly correlated with the pulmonary CT findings of bronchial wall thickening and atelectasis. When evaluating the swallowing function of disabled children, it may be difficult to assess risk of pneumonia by only examining food aspiration, whereas the presence or absence of saliva aspiration could indicate such a risk. Furthermore, our results suggest the need for new benchmarks to evaluate pneumonia risk, such as the assessment of the type or amount of aspirated matter.

## Conclusions

The results of the present study show that among children, risk assessment of pneumonia based only on the presence or absence of food aspiration detected by swallowing function tests may miss significant saliva and secretion aspiration. Moreover, the status of saliva aspiration may be an indicator of aspiration pneumonia risk and may be useful in the management of dysphagia.

## Abbreviations

CT: computed tomography
FEES: fiberoptic endoscopic evaluation of swallowing
PAS: penetration-aspiration scale
VFSS: videofluoroscopic swallowing study

## References

[1] Arvedson JC (2013). Feeding children with cerebral palsy and swallowing difficulties. Eur J Clin Nutr.

[2] Benfer KA, Weir KA, Bell KL, Ware RS, Davies PS, Boyd RN (2013). Oropharyngeal dysphagia and gross motor skills in children with cerebral palsy. Pediatrics.

[3] Sullivan PB, Lambert B, Rose M, Ford-Adams M, Johnson A, Griffiths P (2000). Prevalence and severity of feeding and nutritional problems in children with neurological impairment: Oxford feeding study. Dev Med Child Neurol.

[4] Calis EA, Veugelers R, Sheppard JJ, Tibboel D, Evenhuis HM, Penning C (2008). Dysphagia in children with severe generalized cerebral palsy and intellectual disability. Dev Med Child Neurol.

[5] Weir KA, McMahon S, Taylor S, Chang AB (2011). Oropharyngeal aspiration and silent aspiration in children. Chest.

[6] Owayed AF, Campbell DM, Wang EE (2000). Underlying causes of recurrent pneumonia in children. Arch Pediatr Adolesc Med.

[7] Taniguchi MH, Moyer RS (1994). Assessment of risk factors for pneumonia in dysphagic children: significance of videofluoroscopic swallowing evaluation. Dev Med Child Neurol.

[8] Vaughan D, Katkin JP (2002). Chronic and recurrent pneumonias in children. Semin Respir Infect.

[9] Serel Arslan S, Demir N, Karaduman AA (2018). Both pharyngeal and esophageal phases of swallowing are associated with recurrent pneumonia in pediatric patients. Clin Respir J.

[10] Calvo I, Conway A, Henriques F, Walshe M (2016). Diagnostic accuracy of the clinical feeding evaluation in detecting aspiration in children: a systematic review. Dev Med Child Neurol.

[11] Logemann JA (1986). Treatment for aspiration related to dysphagia: an overview. Dysphagia.

[12] Langmore SE, Terpenning MS, Schork A, Chen Y, Murray JT, Lopatin D, Loesche WJ (1998). Predictors of aspiration pneumonia: how important is dysphagia?. Dysphagia.

[13] Pikus L, Levine MS, Yang YX, Rubesin SE, Katzka DA, Laufer I (2003). Videofluoroscopic studies of swallowing dysfunction and the relative risk of pneumonia. AJR Am J Roentgenol.

[14] Butler SG, Clark H, Baginski SG, Todd JT, Lintzenich C, Leng X (2014). Computed tomography pulmonary findings in healthy older adult aspirators versus nonaspirators. Laryngoscope.

[15] Weir K, McMahon S, Chang AB. Restriction of oral intake of water for aspiration lung disease in children. Cochrane Database Syst Rev. 2012:CD005303. 10.1002/14651858.CD005303.pub3 PMC7389607 22972084

[16] Langmore SE, Schatz K, Olsen N (1988). Fiberoptic endoscopic examination of swallowing safety: a new procedure. Dysphagia.

[17] Langmore SE, Schatz K, Olson N (1991). Endoscopic and videofluoroscopic evaluations of swallowing and aspiration. Ann Otol Rhinol Laryngol.

[18] Rosenbek JC, Robbins JA, Roecker EB, Coyle JL, Wood JL (1996). A penetration-aspiration scale. Dysphagia.

[19] Rao N, Brady SL, Chaudhuri G, Donzelli JJ, Wesling MW (2003). Gold-standard? Analysis of the videofluoroscopic and fiberoptic endoscopic swallow examinations. J Appl Res.

[20] Kelly AM, Leslie P, Beale T, Payten C, Drinnan MJ (2006). Fibreoptic endoscopic evaluation of swallowing and videofluoroscopy: does examination type influence perception of pharyngeal residue severity?. Clin Otolaryngol.

[21] Hartnick CJ, Hartley BE, Miller C, Willging JP (2000). Pediatric fiberoptic endoscopic evaluation of swallowing. Ann Otol Rhinol Laryngol..

[22] Leder SB, Karas DE (2000). Fiberoptic endoscopic evaluation of swallowing in the pediatric population. Laryngoscope.

[23] da Silva AP, Lubianca Neto JF, Santoro PP (2010). Comparison between videofluoroscopy and endoscopic evaluation of swallowing for the diagnosis of dysphagia in children. Otolaryngol Head Neck Surg.

[24] Weir K, McMahon S, Barry L, Ware R, Masters IB, Chang AB (2007). Oropharyngeal aspiration and pneumonia in children. Pediatr Pulmonol.

[25] Cass H, Wallis C, Ryan M, Reilly S, McHugh K (2005). Assessing pulmonary consequences of dysphagia in children with neurological disabilities: when to intervene. Dev Med Child Neurol.

[26] Hiller N, Simanovsky N, Bahagon C, Bogot N, Maayan C (2009). Chest computed tomography findings in familial dysautonomia patients: a model for aspiration. Isr Med Assoc J.

[27] Link DT, Willging JP, Miller CK, Cotton RT, Rudolph CD (2000). Pediatric laryngopharyngeal sensory testing during flexible endoscopic evaluation of swallowing: feasible and correlative. Ann Otol Rhinol Laryngol..

[28] Murray J, Langmore SE, Ginsberg S, Dostie A (1996). The significance of accumulated oropharyngeal secretions and swallowing frequency in predicting aspiration. Dysphagia.

